# Evaluating FDM Process Parameter Sensitive Mechanical Performance of Elastomers at Various Strain Rates of Loading

**DOI:** 10.3390/ma13143202

**Published:** 2020-07-18

**Authors:** Muhammad Salman Chaudhry, Aleksander Czekanski

**Affiliations:** 1 Department of Earth and Space Science, York University, Toronto, ON M3J 1P3, Canada; salman.chaudhry@hotmail.com; 2 Department of Mechanical Engineering, York University, Toronto, ON M3J 1P3, Canada

**Keywords:** FDM, high strain rate, Kolsky bar

## Abstract

To optimize the mechanical performance of fused deposition modelling (FDM) fabricated parts, it is necessary to evaluate the influence of process parameters on the resulting mechanical performance. The main focus of the study was to characterize the influence of the initial process parameters on the mechanical performance of thermoplastic polyurethane under a quasi-static and high strain rate (~2500 s^−1^). The effects of infill percentage, layer height, and raster orientation on the mechanical properties of an FDM-fabricated part were evaluated. At a quasi-static rate of loading, layer height was found to be the most significant factor (36.5% enhancement in tensile strength). As the layer height of the sample increased from 0.1 to 0.4 mm, the resulting tensile strength sample was decreased by 36.5%. At a high-strain rate of loading, infill percentage was found to be the most critical factor influencing the mechanical strength of the sample (12.4% enhancement of compressive strength at 100% as compared to 80% infill). Furthermore, statistical analysis revealed the presence of significant interactions between the input parameters. Finally, using an artificial neural networking approach, we evaluated a regression model that related the process parameters (input factors) to the resulting strength of the samples.

## 1. Introduction

Additive manufacturing (AM), or 3D printing, has transformed various industries, from fabricating custom prototypes to large-scale manufacturing of final products. The continuous development of novel 3D printing techniques and materials is set to revolutionize the way nearly all products are manufactured [1]. This technology offers solutions with a high degree of flexibility and customization in design, top-notch precision, excellent reproducibility, and rapid microfabrication. 3D printed products have been used extensively in product development cycles to test kinematic functionality and for design verification. The aerospace [2] and medical [3,4] industries are also exploiting the advantages offered by AM as it outgrows far beyond its roots in rapid prototyping.

**Figure 1:**
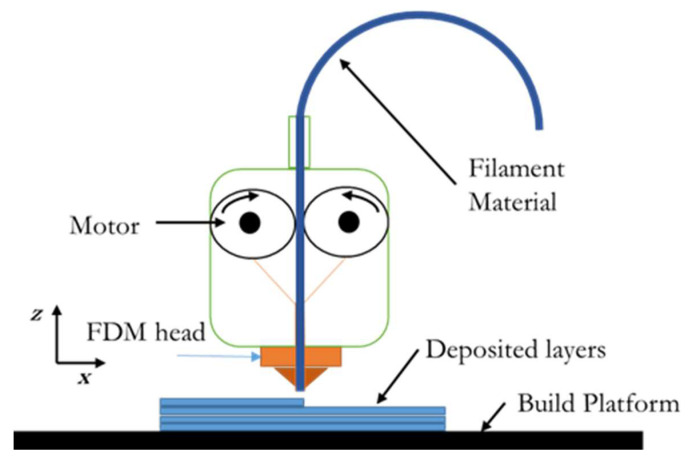
Fused deposition modelling (FDM) process.

Fused deposition modelling, or FDM (3D printing technology as defined by ISO/ASTM 52900), is a process whereby a thermoplastic material is extruded in layers to create a 3D object. In this process, the design of the product is first realized using computer-aided design (CAD) software. From a solid model, the design is exported to the STL tessellated file format. This faceted model is then sliced into parallel horizontal cross-sections. During this process, the build material used (e.g., acrylonitrile butadiene styrene (ABS)) is in the form of a filament [5]. Through a temperature-controlled extrusion nozzle, each layer is melted and deposited onto the build plate in the form of fibres (Figure 1). The finished 3D part takes the form of a vertically stacked laminated composite with a network of fibres and voids. Bonding between adjacent fibres is achieved by thermally driven diffusion welding [6]. Overall bond strength is significantly affected by the envelope temperature and variations in convective conditions within the building chamber [7]. During fabrication, the bottom layers of a part are susceptible to deformation due to residual stresses caused by uneven rapid heating and cooling cycles. This localized contraction and expansion not only affects the mechanical performance of a part but also causes distortions during processing [8]. Residual stress-induced deformation depends on stacking section length [9].

**Figure 2:**
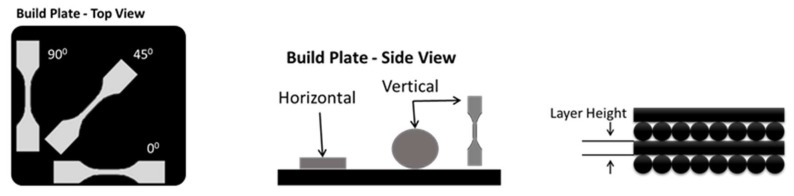
Visualization of sample orientation and layer height.

**Figure 3:**
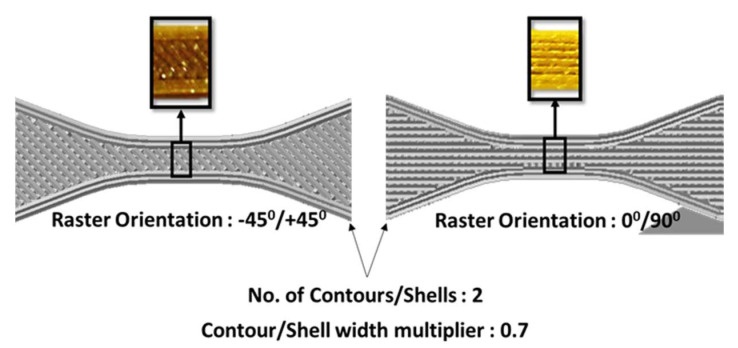
Different raster angle as a result of printing a thermoplastic polyurethane part in different orientations.

FDM produces parts with unique characteristics. The material is deposited in a way that results in anisotropic behaviour owing to the different arrangement of fibres and printing direction. Moreover, the overall quality and performance of a 3D printed part is also affected by the build parameters, namely printing direction (raster angle), infill percentage, and layer thickness (Figure 2 and Figure 3). Recently, many researchers have focused on characterizing how printing process parameters influence final product performance in polymer and metallic 3D printing [10,11,12,13,14,15,16]. Rayegani and Onwubolu [17] modelled a functional relationship between build parameters and tensile strength using the group method of data handling (GMDH). The predicted values were a close match for samples with different orientations and build parameters. A similar study by Sood et al. [6] investigated the influence of layer thickness, raster angles, infill width, and air voids on the bonding and distortion within a part and its mesostructural configuration. They also investigated the effect of these parameters on the compressive strength of the specimens and drew conclusions about the importance of partial debonding in the fibres, which causes buckling [18]. Compressive strength is also affected by anisotropic behaviour, which is influenced by build direction; compressive strength is reduced in the transverse relative to the axial direction [19].

The most common thermoplastic polymers used in FDM-based 3D printers are polylactic acid (PLA) and ABS. With the rapid growth of the consumer-based 3D printing industry, new advanced materials for this process are being developed. One of the most interesting additions to the family of FDM-based printing materials is thermoplastic polyurethane (TPU), which demonstrates high flexibility and rubber-like characteristics. While much attention has been devoted to understanding the impact of process parameters on the performance of popular polymers like ABS, little work has been done to characterize and optimize the process parameters for TPU and other new FDM materials. In terms of the mechanical properties of materials, research has focused thus far on testing.

3D printed parts under quasi-static conditions, neglecting to determine how process parameters affect mechanical properties of parts under a high strain rate versus a quasi-static load.

The aim of this work was to conduct a systematic study of how manufacturing parameters influence the mechanical properties of 3D printed TPU. The process parameters measured were infill percentage, layer height, and raster orientation. Samples were tested for uniaxial tensile strength at a quasi-static loading rate and compressive strength at a high strain rate of loading. In the reported literature, testing has been carried out under quasi-static loading conditions [17,18,19,20,21,22], and the response at high strain has been ignored. Commercially available load frames cannot generate high strain rate loading, and specialized testing equipment is required. An important aspect that this study covers is the performance of build parameters under high strain rate impact-like conditions. The complex and tangled molecular structure of elastomers interacts with loading in a relatively complex way that is sensitive to the rate of loading applied. As an example, elastomers can vary from brittle and glassy at a high strain rate and low temperature to rubbery and flowy at a low strain rate and high temperature.

As the 3D printing industry moves from rapid prototyping to rapid manufacturing, it is essential to characterize and understand the effect of process parameters on parts subjected to different loading conditions in order to maintain overall system integrity and performance. This study offers a steppingstone to eliminating this knowledge barrier by incorporating 3D printed TPU samples exposed to quasi-static and high strain rates. The outcomes of this study are intended to benefit researchers and engineers, allowing them to design elastomer parts for dynamic applications.

## 2. Materials and Methods

In this study, two build parameters—sample orientation and layer thickness—were considered at different infill percentages, the third build parameter. The TPU material used in this study arrived in the form of filament rolls from ColorFabb NGEN FLEX (Belfeld, Netherlands) (see Table 1 for material properties). The flexible filament is comprised of TPU resin containing 99% polyester and <1% color additive (by weight). This material possesses desirable mechanical properties, such as high flexibility and high elongation at break point, compared with traditional FDM materials. A commercial entry-level FDM-based 3D printer was used to fabricate the samples (Prusa Research MK2). Some modifications were made to the extruder to allow it to work with the flexible filament by using custom profile settings. The extrusion temperature along with some other parameters were kept constant while samples were printed with different infill percentages, layer heights, and raster angles (Table 2). The extrusion temperature was kept constant at 230 °C for all the prints and samples.

Quasi-static tension tests were conducted using a commercial load frame (MTS Criterion 43, Eden Prairie, MN, USA) in accordance with ASTM D638 Standard Test Methods for Tensile Properties of Plastic [23]. The tests were carried out at a crosshead speed of 0.05 mm/min, and data were collected at 50 Hz. Extension values were recorded using an MTS Laser Extensometer having an accuracy up to 0.001 mm. From the extension and load values recorded from the tension test, stress–strain curves were generated for further analysis.

**Figure 4:**
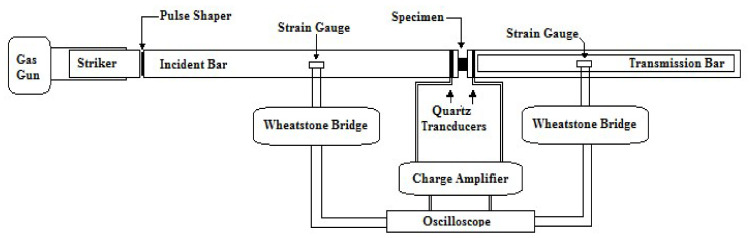
Schematic of the Kolsky bar apparatus used for high-strain rate testing of TPU samples.

To test samples at high strain rates, a Kolsky bar (also known as a split Hopkinson bar) was developed, as commercially available load frames were not able to achieve impact-like conditions. This is the most common apparatus used to test at strain rates of 100 to 10000 s^−1^ [24]. The principal of the Kolsky bar depends on one-way elastic compressive wave propagation through the bar. Samples are fixed between two cylindrical metallic bars, and a striker is accelerated and impacted at one end to generate loading. This incident wave travels through the incident bar, and upon reaching the sample–incident interface, some part of the wave is reflected back, while the rest is transmitted through the specimen to the transmitter bar (Figure 4). Strain histories are recorded in the incident and transmitted bar using strain gauges in a full bridge configuration (to compensate for bending and temperature).

One important consideration while testing samples with a Kolsky bar is that the bars must be concentrically aligned before running the tests; otherwise, the bars create noise in the recorded signals and can compromise the quality of the results. This apparatus is usually used to test high-strength materials like metals; to test softer samples, some modifications to the system are required. Due to a major impedance mismatch between the reflecting boundaries, a smaller-amplitude wave is transmitted to the transmitter bar. Using a hollow transmitter bar can increase the transmitted amplitude greatly due to amplification caused by the cross-sectional area mismatch between the bars. Copper pulse shapers were attached between the striker and the incident bar to (1) reduce the wave dispersion by physically filtering out the high-frequency components in the generated pulse through elastic–plastic deformation and (2) facilitate stress equilibrium by increasing the rise time of the loading pulse. By using a pulse shaper, various profiles of loading pulses can be generated to characterize materials like elastomers, which have characteristics that are affected by the loading shape of the pulse. For testing purposes, the specimen geometry was selected to be *D*_s_ = 12 mm and *L*_s_ = 2.5 mm. The length and the diameter of the bars were selected to be *L*_b_ = 2000 mm and *D*_b_ = 20 mm, respectively (hollow transmitter tube with thickness of 2 mm). The bars were made of aluminum AL6063, and the pulse shaper was copper CU11000. The striker was accelerated using a pressurized gas chamber. The schematic of the setup is illustrated in Figure 4. The detailed design of such a testing system and parameter optimization process can be found in earlier works of the authors [25,26].

## 3. Results

### 3.1. Quasi-Static Tensile Tests

**Figure 5:**
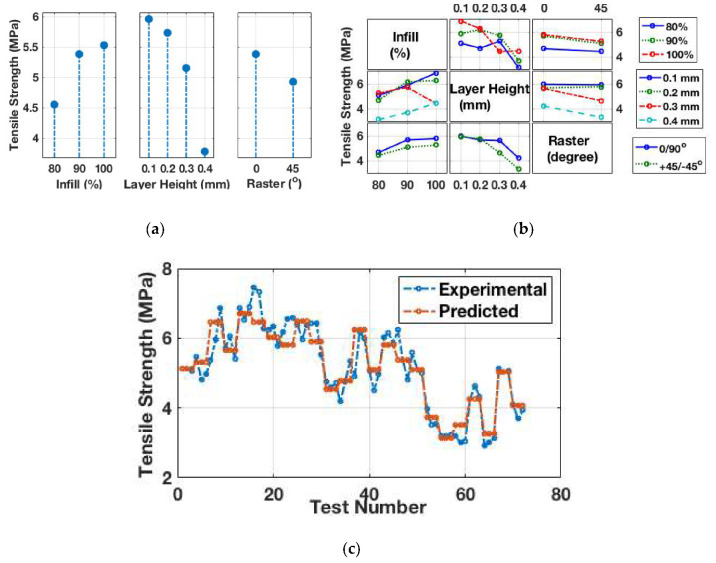
(**a**) Main effect plot of each factor for the quasi-static tensile tests. (**b**) Interaction plot for quasi-static tensile test results. (**c**) Experimental versus predicted tensile strength.

To carry out quasi-static tensile tests, a full factorial approach was applied to the design of the experiment (DOE). This methodology allows a researcher to account for every possible combination of parameters. Table 3 lists the outcomes of three test runs for each possible combination of process parameters. Figure 5a illustrates the categorical mean tensile strength for each of the build parameters at different levels. The main effect plot shows that the most significant factor affecting the tensile strength of 3D printed TPU samples is layer height. As the layer height of the sample increased from 0.1 to 0.4 mm, the resulting tensile strength of the TPU sample decreased by 36.5%. The inverse proportional relation observed between layer height and tensile strength is consistent with some other works [17]. For every 0.1 mm increase in layer height, the tensile strength of the specimen decreased by ~4%, ~10%, and ~27%, respectively. At a layer height of 0.1 mm, due to a greater number of layers in the sample, there is an increased number of load-bearing fibre layers providing a greater area of fibre-to-fibre contact. This results in enhanced micro-reinforcements to the sample. In a layer that is 0.4 mm thick, the distortional effect increases owing to an uneven temperature gradient along the build axis.

Layer height can be viewed as a parameter that enhances the mechanical strength and the overall surface smoothness and finish of 3D printed parts. Figure 5a illustrates that the raster angle, or the arrangement of fibres in the sample, also affects the tensile strength of the specimen, with a reduction of almost 10% in tensile strength when the fibres are rotated from a 0°/90° to a +45°/−45° orientation. At a 0°/90° raster orientation, some of the fibres are aligned in the loading direction. The sample fails when the fibres carrying the load break. At a raster orientation of +45°/−45°, the sample breaks when the thermally fused bonds between the fibres fail. These finding are consistent with the results published in [27,28] (who reported a maximum tensile strength at 0° followed by 45° and then 90°), but not with [29] (who reported a 45° raster orientation to achieve higher tensile strength). Overall, raster orientation was shown to be less sensitive than layer height to tensile strength in samples (consistent with findings in [17]). Furthermore, it is interesting to see an increase in the tensile strength as the infill percentage in the samples increases. Strengthening is reduced from 90% infill to 100%. The probable reason for this behaviour can be determined by considering the thermal degradation of material due to the presence of a heated extruder head for an extended period of time on a region with high infill. Infill percentage at 100% results in no or negative air gaps between the adjacent layers, leading to restricted heat dissipation and the accumulation of extra stress. This resulted in a ~2.7% increase in tensile strength, from 5.38 MPa at 90% infill to 5.53 MPa at 100% infill, compared with an 18.1% increase in tensile strength from 80% infill to 90%. The proportional effect of infill percentage on tensile strength is consistent with other works [30]. Furthermore, a possible interaction between the processing parameters can be deduced from the interaction plot. Figure 5b reveals a visible interaction between the height and infill percentage in the sample. It is also evident that raster angle has no combined effect with either the layer height or infill percentage in the sample.

In the next part of this study, an artificial neural network (ANN) model was built in MATLAB, with the levels of the printing process parameters used as the input and the experimental tensile strength as the output. Examples of the application have been described in [19,20]. The ANN model was trained with the Levenberg–Marquardt algorithm and consisted of three inputs, 10 tan–sigmoid activated neurons in the hidden layer, and one linear output neuron in the output layer. The test data, presented in Table 3, were randomly distributed into training, testing, and validation sets. The performance of the model was tracked using mean squared error of the cost function and the correlation between the output and target value. Figure 5c illustrates the comparison between the experimental and model results. The ANN model provides close agreement and can be used as a valuable predictive tool when dealing with a large number of printing process parameters with multiple levels. However, the ANN modelling process does not provide any information about the effect of each factor on the predicted output tensile strength.

### 3.2. Compressive Response to High Strain Rate

**Figure 6:**
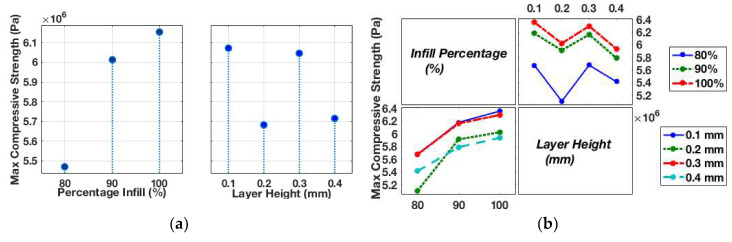

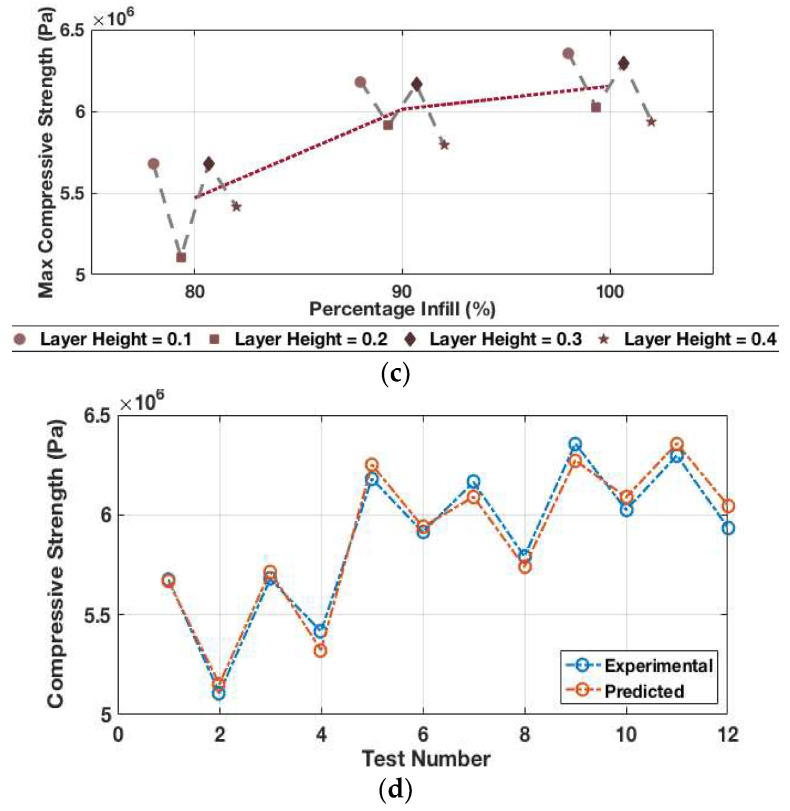
(**a**) Main effect plot of each factor for compression tests at ~2500 s^−1^. (**b**) Interaction plot for high strain compression tests at 2500 s^−1^. (**c**) Multivariate plots for high strain rate compressive tests. (**d**) Experimental versus predicted compressive strength at a constant strain rate of ~2500 s^−1^.

For compression tests conducted at a high strain rate, the factors incorporated in the DOE included infill percentage and layer height. Three different levels for infill percentage (100%, 90%, and 80%) and layer height (0.1, 0.2, 0.3, and 0.4 mm) were considered. For all the tests conducted, the applied strain rate was fixed at ~2500 s^−1^. Table 4 shows the average maximum compressive strength attained in the sample for three test runs using various printing process parameters. First, the printing process parameters and their effect on the compressive strength of samples were studied, as illustrated in Figure 6a. Interestingly, at high strain rates, the printing process parameters and their influence on mechanical strength change the characteristics from what was observed for the quasi-static tensile results. The maximum compressive strength first increases from 5.47 MPa at 80% infill to 6.01 MPa at 90%, and then 6.15 MPa at 100% infill at an increase of only 2.4%. A different behaviour is observed as the layer height in the samples increases. The resulting compressive strength is decreased by ~6.41%, from 6.07 MPa at a 0.1 mm layer height to 5.68 MPa at a 0.2 mm layer height. As the layer height increases from 0.2 to 0.3 mm, the compressive strength increases by 6.41%, followed by 5.71 MPa (a drop of around 5.5%) at a 0.4 mm layer height. At high strain, infill percentage remains the most significant printing parameter affecting compressive strength. In the different layer height scenarios, there appear to be optimum levels that result in samples with the highest compressive strength. These characteristics were not observed for the quasi-static tensile results, or they may not have been captured in the levels considered for each printing parameter. Furthermore, the interaction plot presented in Figure 6b reveals a significant influence of layer height and infill interaction, which is more evident in the multivariate plot illustrated in Figure 6c. The figure presents the variation in compressive strength at different layer heights for different infill percentages. The variation due to layer height at 80% is similar to the variation at both 90% and 100% infill.

Next, an ANN model was developed for compressive test runs at a high strain rate. An ANN model trained with the Levenberg–Marquardt algorithm consisted of 10 neurons in the hidden layer and one neuron in the output layer. The neural network was allocated 70% of the test data for training purposes, 15% to validation, and 15% to testing. The predicted values are in good agreement with the experimental values, indicating that this approach would be useful for modelling the mechanical strength of 3D printed parts (Figure 5c and Figure 6d).

## 4. Conclusions

In this study, we investigated the effect of 3D printing process parameters on the mechanical tensile strength of TPU samples under both quasi-static conditions and maximum compressive strength at a high strain rate (2500 s^−1^). This included analyzing how infill percentage, layer height, and raster angle affected the mechanical properties of the TPU samples and the interactions between these factors. Our results offer a good idea of how different values of infill percentage, raster orientation, and layer height respond to quasi-static tensile strength and compressive strength at a high strain rate. Our results revealed layer height to be the most statistically significant factor affecting the performance of the sample under quasi-static loading. Contrary to this finding, under a high strain rate of loading, infill percentage was found to be the most critical factor influencing the mechanical strength of the sample. Furthermore, statistical analysis revealed the relative importance and presence of significant interactions between the input parameters. To fully understand and characterize this relation, a wide range of other process parameters at many different levels need to be considered, such as nozzle speed and lower values of infill percentage, so that valid conclusions can be reached.

## Figures and Tables

**Table 1:** Material properties for thermoplastic polyurethane (TPU) flexible filament as received from the supplier.

Tensile Strength Yield	Tensile Strength Ultimate	Hardness	Melting Point	Specific Gravity	Moisture Absorption
14 MPa	22 MPa	55 shore D	250 °C	1.13 g/cc	0.22%

**Table 2:** The 3D printing process parameters of thermoplastic urethane samples.

Control Factors			Fixed Factors	
	Quasi-Static	High-Strain Rate		
Percentage Infill (%)	100	100	Part Orientation No. of Shells Nozzle Diameter Coarseness Part OrientationNozzle temperatureHeated bedOpen/closed chamber	Flat 2 0.4 0.0001 mm Horizontal230° noopen
	90	90		
	80	80		
Layer Height (mm)	0.1	0.2 0.3 0.4		
	0.2			
	0.3			
	0.4			
Raster Angle (°)	0/90 45/45	0/90		

**Table 3:** Input process parameter and corresponding experimental and predicted tensile strength.

Printing Parameter			Tensile Strength (MPa)				
Percentage Infill (%)	Layer Height (µm)	Raster Angle (°)	Test 1	Test 2	Test 3	Mean	Predicted
80	0.1	0/90	5.13	5.11	5.07	5.10	5.12
80	0.1	+45/−45	5.46	4.81	4.96	5.08	5.31
80	0.2	0/90	4.76	4.58	4.71	4.68	6.47
80	0.2	+45/−45	4.18	4.74	5.34	4.76	5.67
80	0.3	0/90	4.89	6.17	6.00	5.69	6.71
80	0.3	+45/−45	5.07	4.51	4.97	4.85	6.47
80	0.4	0/90	3.21	3.19	3.24	3.21	6.01
80	0.4	+45/−45	3.21	3.00	3.03	3.08	5.81
90	0.1	0/90	5.37	5.97	6.88	6.07	6.49
90	0.1	+45/−45	5.70	6.05	5.41	5.72	5.92
90	0.2	0/90	6.41	5.97	6.38	6.25	4.52
90	0.2	+45/−45	6.42	6.43	5.53	6.13	4.77
90	0.3	0/90	6.01	6.16	5.84	6.00	6.25
90	0.3	+45/−45	6.23	5.39	4.81	5.47	5.08
90	0.4	0/90	4.24	4.64	4.33	4.40	5.81
90	0.4	+45/−45	2.90	3.01	3.13	3.02	5.38
100	0.1	0/90	6.86	6.54	6.89	6.76	5.10
100	0.1	+45/−45	7.47	7.32	6.27	7.02	3.74
100	0.2	0/90	6.23	6.34	5.77	6.12	3.14
100	0.2	+45/−45	6.20	6.57	6.60	6.45	3.50
100	0.3	0/90	5.58	5.10	4.99	5.22	4.26
100	0.3	+45/−45	3.99	3.50	3.53	3.67	3.24
100	0.4	0/90	5.14	5.02	5.05	5.07	5.02
100	0.4	+45/−45	4.10	3.68	3.93	3.90	4.07

**Table 4:** Input process parameter and resulting experimental and predicted compressive strength at a high strain rate.

Printing Parameter		Compressive Strength (Mpa)	
Percentage Infill (%)	Layer Height (mm)	Measured	Predicted
80	0.1	5.68	5.67
80	0.2	5.11	5.15
80	0.3	5.68	5.72
80	0.4	5.42	5.32
90	0.1	6.18	6.25
90	0.2	5.91	5.94
90	0.3	6.16	6.09
90	0.4	5.79	5.74
100	0.1	6.36	6.27
100	0.2	6.02	6.09
100	0.3	6.30	6.36
100	0.4	5.94	6.05
